# Heavy metal transport with adsorption for instantaneous and exponential attenuation of concentration

**DOI:** 10.1038/s41598-023-50754-5

**Published:** 2024-01-04

**Authors:** Ruishi Liang, Z. M. Isa

**Affiliations:** 1 School of Mathematics and Statistics Shaoguan University, Shaoguan, 512005 Guangdong China; 2 Department of Mathematical Sciences, Faculty of Science, Universiti Teknologi Malaysia, Johor Bahru, 81310 Johor Malaysia; 3 UTM-Centre for Industrial and Applied Mathematics (UTM-CIAM), Ibnu Sina Institute for Scientific and Industrial Research, Universiti Teknologi Malaysia, Johor Bahru, 81310 Johor Malaysia

**Keywords:** Environmental sciences, Mathematics and computing, Physics

## Abstract

Heavy metal pollutant is a serious problem in environmental pollution, and it is very difficult to eradicate once it enters the soil. As heavy metal adsorption has been proven to occur, the heavy metal’s behaviour can be modeled as a transport equation with adsorption. Previous adsorption term mostly due to the concentration alone, while in here, the desorption effect given by the rate of change of the concentration is also included. Also, the heavy metals are frequently considered to enter the soil after being dumped into the soil for a certain period of time. But, quick dumping onto the soil can introduce heavy metal instantaneously. Heavy metals entering the soil through leaching or when their concentration in the soil is influenced by chemical reactions, can all lead to the exponential decay of heavy metals entering the soil. Based on two-dimensional advection diffusion equation (ADE) with the new adsorption term, analytical solutions are obtained for the cases of instantaneous and exponential attenuation of heavy metals emission to soil by the method of Laplace transform. The results highlight the significant influence of emission type on the peak concentrations. If heavy metals are instantaneously enter the soil, the peak occurs in the range of 1–3 m radius from the point of emission on the first day, while for exponential attenuation the peak occurs close to the point of emission. Furthermore, there exists a correlation between retardation factors and heavy metal concentrations, where a decrease in retardation factors leads to an increase in heavy metal concentration. It is essential to investigate both types of heavy metals emission to provide valuable information for proper pollution management, effective environmental regulations and enforcement.

## Introduction

Heavy metal pollution is a serious environmental pollution because it is a kind of persistent pollutant in soil environment. Heavy metals enter the soil through atmospheric deposition, industrial emissions, or waste accumulation, and are highly toxic, posing a significant threat to crops, plants, and soil microorganisms.^1^ Researching the migration and characteristics of heavy metals in soil to provide essential support for environmental scientists is crucial. Where research in this direction, has included the analytical solutions of ADEs.

In the study of analytical solutions for the ADE governing the migration of heavy metals in soil, the adsorption term is a highly significant factor. Such as Chai et al.^2^ considered the adsorption of heavy metals under the action of activated carbon surface adsorbents and Senthikumar et al.^3^ believed that biological adsorbents should fully consider conditions such as PH value and temperature. Al-ghouti et al.^4^ believed that the simplest adsorption is that the adsorption constant is proportional to the surface adsorbent which is supported by Shuai et al.^5^. They incorporated this adsorption term into the ADE and obtained analytical solutions; unfortunately, the equation only considered adsorption equilibrium and non-equilibrium in one-dimensional direction. In summary, these researchers’ studies on the adsorption term only accounted for a single direction, which is not applicable to most real-world scenarios. Adsorption is not only spatially dependent but also time-dependent over time. Therefore, adding a time-dependent component to the adsorption term is necessary.

Instantaneous emission refers to the emission or introduction of heavy metals within an extremely short period. While exponential decay signifies a gradual released over a certain period. Initially, the concentration is relatively high and then decreases exponentially over time. In studies related to pollutant emission, researchers often consider various boundary conditions to provide a comprehensive explanation. For example, Chaudhary et al.^6^ employed Dirichlet-type and Neumann-type boundary conditions to describe the migration of pollutants in aquatic environments. However, their research is limited to one-dimensional cases and in aquatic environments, pollutant migration is relatively straightforward. This approach may not suffice for the migration of heavy metals in soil, as specific heavy metals require specialized treatment, reducing time wastage. Similarly, Bharati et al.^7^ also investigated the analytical solutions for one-dimensional transient ADEs, incorporating numerical solutions to complement their analytical results. While this research is highly relevant for pollutant migration in one-dimensional hydrological environments, it may not be applicable to soil environments, which are considerably more complex, particularly regarding the migration of heavy metals. In such cases, two-dimensional or higher-dimensional models become more appropriate. Some researchers have considered both instantaneous and exponential decay emission scenarios, such as the study conducted by Aral et al.^8^. Nevertheless, the absence of adsorption terms in their models is insufficient for comprehensively addressing the migration of heavy metals in soil. Abhishek^9^ primarily focused on the migration of pollutants from instantaneous point sources and continuous point sources in groundwater and rivers, obtaining analytical solutions using the Green’s function method. Similarly, this approach is feasible; however, it is limited to one-dimensional scenarios and migration within the aquatic environment, making it unsuitable for the transport of heavy metals through porous media such as soil layers. Yang et al.^5^ and Garcia et al.^10^ proposed pollutants transport in porous media in ADE with adsorption. The model only considered a constant boundary condition and for one-dimensional only. The work by Chen et al.^11^ studied the migration of pollutants in unsaturated porous media by considering analytical solutions for both instantaneous emission and exponential decay of concentration. Their findings were based solely on one-dimensional advection-diffusion adsorption numerical simulations.

There is limited usage of the two-dimensional ADE to describe the migration of heavy metals in soil in the literature. Yadav et al.^12^ considered the two-dimensional ADE to describe the migration model of pollutants in soil, but they overlooked the importance of adsorption. Mustafa et al.^8^ developed instantaneous and continuous point source solutions under the conditions of a two-dimensional ADE with constant, linear, asymptotic, and exponential varying diffusion coefficients. They also neglected the role of adsorption. Batu et al.^13^ and Fedi et al.^14^ have used Laplace or Fourier transform to get the analytical solution for two-dimensional ADE which showed the pollutants transport in the porous media. None of the ADE models have included adsorption with instantaneous emission and exponential attenuation of concentration emission. Thakur et al.^15^ discussed a variety of boundary conditions, homogeneous and heterogeneous media, instantaneous flow. While exploring diverse boundary conditions can be valuable for research, it also introduces complexity. It may make less applicable to practitioners who require straightforward.

This article primarily discusses the migration of heavy metals in soil within the context of a two-dimensional ADE. It underscores that the adsorption term is a critical factor, which not only exhibits spatial dependence but also temporal variation. These attributes arise because the adsorption term involves both adsorption and desorption processes during the migration of heavy metals in the soil. Furthermore, within the boundary conditions, two different emission forms are introduced: instantaneous emission and exponential decay emission of concentration. These forms encompass scenarios where heavy metals are accidentally released into the soil for extremely short time and where they remain in the soil for an extended period due to various chemical reactions, PH levels, and blocking factors. Over time and distance, the concentration exhibits exponential decay. Consequently, the three main points discussed in this paper namely, the adsorption term, instantaneous emission, and exponential decay emission of concentration can effectively describe most situations related to the migration of heavy metals in the soil. This research provides valuable guidance for environmental practitioners dealing with such issues.

## Governing equations

Under the condition of two-dimensional equation, the heavy metal migration with adsorption satisfies the partial differential equation describing advection–diffusion in porous media such that^13,16^

1 $$\begin{aligned} R\frac{\partial C}{\partial t}=D_x\frac{\partial ^2 C}{\partial x^2}+D_y\frac{\partial ^2 C}{\partial y^2}-u\frac{\partial C}{\partial x}-v\frac{\partial C}{\partial y}-\frac{\rho }{\theta }\frac{\partial S}{\partial t}, \end{aligned}$$

the $\frac{\rho }{\theta }\frac{\partial S}{\partial t}$ is the adsorption term. *R* is the retardation factor, *C* $[ML^{-3}]$ is the concentration of heavy metal ions in seepage, *S* is the adsorption concentration; $\theta$ is the porosity of porous media; *u* $[LT^{-1}]$ is the uniform seepage velocity along *x* or longitudinal direction, *v* $[LT^{-1}]$ is the uniform seepage velocity along *y* or transverse direction, *t* [*T*] is time, *x*, *y* [*L*] are the migration distance of heavy metal particles, $\rho$ $[ML^{-3}]$ is the particle density, $D_x$ and $D_y$ $[L^2T^{-1}]$ are diffusion coefficient along longitudinal or transverse direction respectively. The relationship between the diffusion rate and the concentration of adsorbed material in space and the change rate of adsorbed material with time are considered. The adsorption term is

2 $$\begin{aligned} \frac{\rho }{\theta }\frac{\partial S}{\partial t}=k_xC(x,y,t)-k_y\frac{\partial C}{\partial t}, \end{aligned}$$

here $k_x$ is adsorption rate coefficient and $k_y$ is desorption rate coefficients through *x* and *y*. Therefore, Eq. (1) becomes

3 $$\begin{aligned} (R-k_y)\frac{\partial C}{\partial t}=D_x\frac{\partial ^2 C}{\partial x^2}+D_y\frac{\partial ^2 C}{\partial y^2}-u\frac{\partial C}{\partial x}-v\frac{\partial C}{\partial y}-k_xC. \end{aligned}$$

Furthermore, the diffusion coefficient is considered to be geometrically proportional to the seepage velocity^17–19^, namely

4 $$\begin{aligned} D_x=au\ \ \text {and} \ \ D_y=bv, \end{aligned}$$

here *a* and *b* are the coefficients that depends upon pore geometry and average pore size diameter of the porous media. Two types of heavy metal types of emission are considered in this paper. Therefore, the following boundary conditions are separated into instantaneous emission and exponential attenuation of concentration.

### Instantaneous emission

In this case, the heavy metal is instantaneously introduced to the porous medium (soil) . Typically taken to be a uniform pulse type point source. It is assumed $C_i$ heavy metal concentration initially and flux type conditions are assumed at infinite domain. Assuming that the heavy metal instantaneously enters the domain at the point (0,0), hence, the corresponding initial and boundary conditions are

5 $$\begin{aligned}{} & {} C(x,y,0)=C_{i},\quad 0\le x<\infty , \quad 0\le y <\infty , \end{aligned}$$

6 $$\begin{aligned}{} & {} C(0,0,t)=\frac{m}{Q}\delta (t), \end{aligned}$$

7 $$\begin{aligned}{} & {} \frac{\partial C}{\partial x}=0,\quad \frac{\partial C}{\partial y}=0; \quad x\rightarrow \infty ,y\rightarrow \infty , \end{aligned}$$

where *m* [*M*] is the mass of implanted heavy metal , *Q* is void fraction and $\delta$ $[L^{-2}]$ is the Dirac Delta function.

Let’s introduce a new space variable

8 $$\begin{aligned} z=x+y\sqrt{\frac{D_y}{D_x}}. \end{aligned}$$

Substituting Eq. (8) into Eq. (3) and combining with Eq. (4), yields

9 $$\begin{aligned}{} & {} (R-k_y)\frac{\partial C}{\partial t}=D\frac{\partial ^2 C}{\partial z^2}-U\frac{\partial C}{\partial z}-k_xC, \end{aligned}$$

10 $$\begin{aligned}{} & {} D=D_x(1+\frac{D^{2}_y}{D^{2}_x}),\quad \text {and} \quad U=u+v\sqrt{\frac{bv}{au}}. \end{aligned}$$

Also, based on the new space variable *z*, the initial and boundary conditions of Eqs. (5–7) can be rewritten as

11 $$\begin{aligned}{} & {} C(z,0)=C_{i},\quad 0\le z<+\infty , \end{aligned}$$

12 $$\begin{aligned}{} & {} C(0,t)= \frac{m}{Q}\delta (t), \end{aligned}$$

13 $$\begin{aligned}{} & {} \frac{\partial C(\infty ,t)}{\partial z}=0, \quad z\rightarrow \infty . \end{aligned}$$

In order to solve Eq. (9), Laplace transformation techniques is applied with respect to *t*, which transform Eq. (9) and its initial and boundary conditions Eq. (11–13) into

14 $$\begin{aligned}{} & {} (R-k_y)(s\bar{C}-C_i)=D\frac{\partial ^2 \bar{C}}{\partial z^2}-U\frac{\partial \bar{C}}{\partial z}-k_x\bar{C}, \end{aligned}$$

15 $$\begin{aligned}{} & {} \bar{C}(0,s)=m/Q,\quad \text {and}\quad \frac{\partial \bar{C}(\infty , s)}{\partial z}=0. \end{aligned}$$

The Eq. (14) is a second order ordinary differential equations and the solution can be found of the form,

16 $$\begin{aligned} \bar{C}= & {} I_1+I_2+I_3, \end{aligned}$$

17 $$\begin{aligned} I_1= & {} \frac{m}{Q}\exp \bigg ({\frac{U}{2D}z}\bigg )\exp \bigg ({-z\sqrt{\frac{U^2}{4D^2}+\frac{As-AC_i+k_x}{D}}}\bigg ), \end{aligned}$$

18 $$\begin{aligned} I_2= & {} -\frac{AC_i}{A(s-C_i)+k_x}\exp \bigg ({\frac{U}{2D}z}\bigg )\exp \bigg ({-z\sqrt{\frac{U^2}{4D^2}+\frac{As-AC_i+k_x}{D}}}\bigg ), \end{aligned}$$

19 $$\begin{aligned} I_3= & {} \frac{AC_i}{A(s-C_i)+k_x}, \end{aligned}$$

where $A=R-k_y$. Application of the inverse laplace transform yields

20 $$\begin{aligned} L^{-1}{I_1}= & {} \frac{mz}{2Q\sqrt{\pi \frac{D}{A}t^3}}\exp \bigg (\frac{Uz}{2D}-\frac{z^2}{4\frac{D}{A}t}-\frac{U^2+4ADk_x}{4AD}t+C_it\bigg ), \end{aligned}$$

21 $$\begin{aligned} L^{-1}{I_2}= & {} -\frac{C_i}{2}\exp \bigg (\big (C_i-\frac{k_x}{A}\big )t+\frac{Uz}{2D}\bigg ) \biggl \{\exp \bigg (-z\sqrt{\frac{\frac{U^2}{4D}+(A-1)k_x}{D}}\bigg ){\textrm{erfc}}\bigg (\frac{z\sqrt{A}}{2\sqrt{Dt}}\nonumber \\ {} & {} -\sqrt{\big (\frac{U^2}{4AD}+k_x-\frac{k_x}{A}\big )t}\bigg ) +\exp \bigg (z\sqrt{\frac{\frac{U^2}{4D}+(A-1)k_x}{D}}\bigg )\nonumber \\{} & {} {\textrm{erfc}}\bigg (\frac{z\sqrt{A}}{2\sqrt{Dt}}+\sqrt{\big (\frac{U^2}{4AD}+k_x-\frac{k_x}{A}\big )t}\bigg )\biggr \}, \end{aligned}$$

22 $$\begin{aligned} L^{-1}{I_3}= & {} C_i\exp \bigg (\big (C_i-\frac{k_x}{A}\big )t\bigg ). \end{aligned}$$

Therefore, the desired analytical solution is

23 $$\begin{aligned} \begin{aligned} C_1(z,t)&=\frac{mz}{2Q\sqrt{\pi \frac{D}{A}t^3}}\exp \bigg (\frac{Uz}{2D}-\frac{z^2}{4\frac{D}{A}t}-\frac{U^2+4Dk_x}{4AD}t+C_it\bigg ) -\frac{C_i}{2}\exp \bigg (\big (C_i-\frac{k_x}{A}\big )t+\frac{Uz}{2D}\bigg ) \\ {}&\quad \times \biggl \{\exp \bigg (-z\sqrt{\frac{\frac{U^2}{4D}+(A-1)k_x}{D}}\bigg )\textrm{erfc}\bigg (\frac{z\sqrt{A}}{2\sqrt{Dt}}-\sqrt{\big (\frac{U^2}{4AD}+k_x-\frac{k_x}{A}\big )t}\bigg )\\ {}&\ \quad + \exp \bigg (z\sqrt{\frac{\frac{U^2}{4D}+(A-1)k_x}{D}}\bigg )\textrm{erfc}\bigg (\frac{z\sqrt{A}}{2\sqrt{Dt}}+\sqrt{\big (\frac{U^2}{4AD}+k_x-\frac{k_x}{A}\big )t}\bigg )\biggr \} \\ {}&\quad +C_i\exp \bigg (\bigg (C_i-\frac{k_x}{A}\bigg )t\bigg ). \end{aligned} \end{aligned}$$

When the initial concentration becomes zero, the solution will be reduced to

24 $$\begin{aligned} C_2(z,t)=\frac{mz}{2Q\sqrt{\pi \frac{D}{A}(t)^3}}\exp \bigg (-\frac{z^2}{4\frac{D}{A}t}-\frac{k_x}{A}t\bigg ) \end{aligned}$$

which similar to the problem by Wang et al.^20^ and Mojtabi et al.^21^.

### Exponential attenuation of concentration

In some cases, the emission of heavy metals is proven to decline over time^22,23^, therefore, the exponential attenuation of concentration boundary condition is considered. For this case, heavy metal that is having an exponential decay is introduced at the point (0,0). Therefore, the corresponding initial and boundary conditions are

25 $$\begin{aligned}{} & {} C(x,y,0)=C_i,\quad 0\le x<+\infty , \quad 0\le y<+\infty , \end{aligned}$$

26 $$\begin{aligned}{} & {} C(0,0,t)=C_0e^{-\alpha t}, \end{aligned}$$

27 $$\begin{aligned}{} & {} \frac{\partial C}{\partial x}=0, \quad \frac{\partial C}{\partial y}=0, \quad x\rightarrow \infty , y\rightarrow \infty , \end{aligned}$$

where *C* is the concentration of heavy metal with exponential decay, $\alpha$ is attenuation coefficient of emission concentration, $C_0$ is the initial concentration of injected heavy metal contaminants. By using the same new space variable in Eq. (8), the corresponding initial and boundary conditions now become

28 $$\begin{aligned}{} & {} C(z,0)=C_{i},0\le z<+\infty , \end{aligned}$$

29 $$\begin{aligned}{} & {} C(0,t)=C_0e^{-\alpha t}, \end{aligned}$$

30 $$\begin{aligned}{} & {} \frac{\partial C(\infty ,t)}{\partial z}=0, \quad z\rightarrow \infty . \end{aligned}$$

Therefore, after the necessary working which is similar to previous Eq. (9), the solution can be written of the form

31 $$\begin{aligned} \bar{C}= & {} I_{1}+I_{2}+I_{3}, \end{aligned}$$

32 $$\begin{aligned} I_{1}= & {} \frac{C_0}{s+\alpha }\exp \bigg ({\frac{U}{2D}z}\bigg )\exp \bigg ({-z\sqrt{\frac{U^2}{4D^2}+\frac{As-AC_i+k_x}{D}}}\bigg ), \end{aligned}$$

33 $$\begin{aligned} I_{2}= & {} -\frac{AC_i}{A(s-C_i)+k_x}\exp \bigg ({\frac{U}{2D}z}\bigg )\exp \bigg ({-z\sqrt{\frac{U^2}{4D^2}+\frac{As-AC_i+k_x}{D}}}\bigg ), \end{aligned}$$

34 $$\begin{aligned} I_{3}= & {} \frac{AC_i}{A(s-C_i)+k_x}. \end{aligned}$$

Taking the inverse laplace transform yields

35 $$\begin{aligned} L^{-1}{I_{1}}= & {} \frac{C_0}{2}\exp \bigg (\frac{Uz}{2D}-\alpha t\bigg ) \biggl \{\exp \bigg (-z\sqrt{\frac{\frac{U^2}{4AD}+k_x-C_i-\alpha }{\frac{D}{A}}}\bigg ) \textrm{erfc}\bigg (\frac{z}{2\sqrt{\frac{D}{A}t}}\nonumber \\{} & {} -\sqrt{\big (\frac{U^2}{4AD}+k_x-C_i-\alpha \big )t}\bigg )+ \exp \bigg (z\sqrt{\frac{\frac{U^2}{4AD}+k_x-C_i-\alpha }{\frac{D}{A}}}\bigg ) \textrm{erfc}\bigg (\frac{z}{2\sqrt{\frac{D}{A}t}}\nonumber \\{} & {} +\sqrt{\big (\frac{U^2}{4AD}+k_x-C_i-\alpha \big )t}\bigg ) \biggr \}, \end{aligned}$$

36 $$\begin{aligned} L^{-1}{I_{2}}= & {} -\frac{C_i}{2}\exp \bigg ((C_i-\frac{k_x}{A})t+\frac{Uz}{2D}\bigg ) \biggl \{\exp \bigg (-z\sqrt{\frac{\frac{U^2}{4D}+(A-1)k_x}{D}}\bigg )\textrm{erfc}\bigg (\frac{z\sqrt{A}}{2\sqrt{Dt}}\nonumber \\{} & {} -\sqrt{\big (\frac{U^2}{4AD}+k_x-\frac{k_x}{A}\big )t}\bigg ) +\exp \bigg (z\sqrt{\frac{\frac{U^2}{4D}+(A-1)k_x}{D}}\bigg )\nonumber \\{} & {} \textrm{erfc}\bigg (\frac{z\sqrt{A}}{2\sqrt{Dt}}+\sqrt{\big (\frac{U^2}{4AD}+k_x-\frac{k_x}{A}\big )t}\bigg )\biggr \}, \end{aligned}$$

37 $$\begin{aligned} L^{-1}{I_{3}}= & {} C_i\exp \bigg ((C_i-\frac{k_x}{A})t\bigg ). \end{aligned}$$

Finally, the solution of the concentration *C* is obtained as

38 $$\begin{aligned} \begin{aligned} C&=\frac{C_0}{2}\exp \bigg (\frac{Uz}{2D}-\alpha t\bigg ) \biggl \{\exp \bigg (-z\sqrt{\frac{\frac{U^2}{4AD}+k_x-C_i-\alpha }{\frac{D}{A}}}\bigg ) \textrm{erfc}\bigg (\frac{z}{2\sqrt{\frac{D}{A}t}}-\sqrt{\big (\frac{U^2}{4AD}+k_x-C_i-\alpha \big )t}\bigg )\\&\quad +\exp \bigg (z\sqrt{\frac{\frac{U^2}{4AD}+k_x-C_i-\alpha }{\frac{D}{A}}}\bigg ) \textrm{erfc}\bigg (\frac{z}{2\sqrt{\frac{D}{A}t}}+\sqrt{\big (\frac{U^2}{4AD}+k_x-C_i-\alpha \big )t}\bigg ) \biggr \}\\&\quad -\frac{C_i}{2}\exp \bigg ((C_i-\frac{k_x}{A})t+\frac{Uz}{2D}\bigg )\\&\quad \times \biggl \{\exp \bigg (-z\sqrt{\frac{\frac{U^2}{4D}+(A-1)k_x}{D}}\bigg )\textrm{erfc}\bigg (\frac{z\sqrt{A}}{2\sqrt{Dt}}-\sqrt{\big (\frac{U^2}{4AD}+k_x-\frac{k_x}{A}\big )t}\bigg )\\&\quad +\exp \bigg (z\sqrt{\frac{\frac{U^2}{4D}+(A-1)k_x}{D}}\bigg )\textrm{erfc}\bigg (\frac{z\sqrt{A}}{2\sqrt{Dt}}+\sqrt{\big (\frac{U^2}{4AD}+k_x-\frac{k_x}{A}\big )t}\bigg )\biggr \} +C_i\exp \bigg (\big (C_i-\frac{k_x}{A}\big )t\bigg ). \end{aligned} \end{aligned}$$

## Results and discussion

We have derived analytical solutions for the case of instantaneous emission, which correspond to events such as sudden leaks.The obtained analytical solutions given by Eqs. (23) and (38) permit a wide range of concentration profiles to be examined. In contrast, the exponential decay emission is more applicable to pollution sources with longer durations. The distance (*m*) of vertical and horizontal are taken as $0<x<6$ and $0<y<6$ respectively. Furthermore, we have assessed the varying impact of different emissions on peak concentrations.

### Instantaneous emission

Having obtained the analytical solution in Eq. (23), the two-dimensional contour plot in *x* and *y* can be portrayed. The model parameters are selected by referring to previous studies^11,12,19^ as shown in Table 1.

**Table 1:** Model parameters for instantaneous emission simulations^11,12,19^.

*m*(*mg*)	*Q*(*ml*/*s*)	$k_x (s^{-1})$	$k_y (s^{-1})$	*R*	*a*	*b*	*u*(*cm*/*s*)	*v*(*cm*/*s*)	$C_i (mg/m^3)$
100	5	0.01	0.02	1.5	0.1	0.2	0.75	0.075	0.05

**Figure 1:**
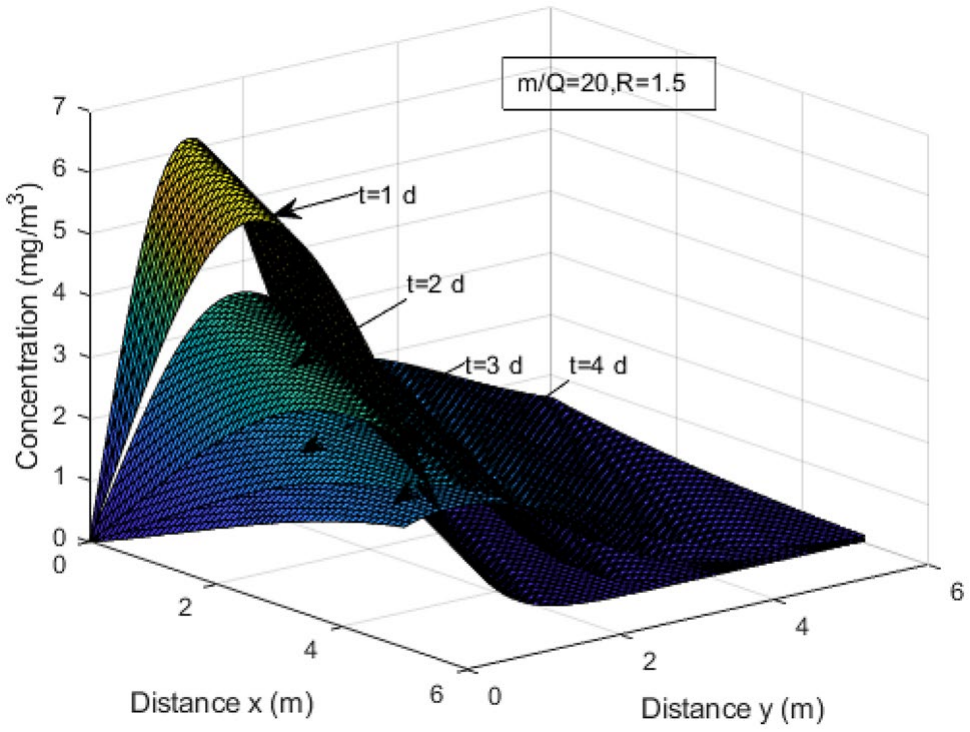
Concentration profiles of heavy metals at different time for fixed *m*/*Q*=20 and *R*=1.5.

**Figure 2:**
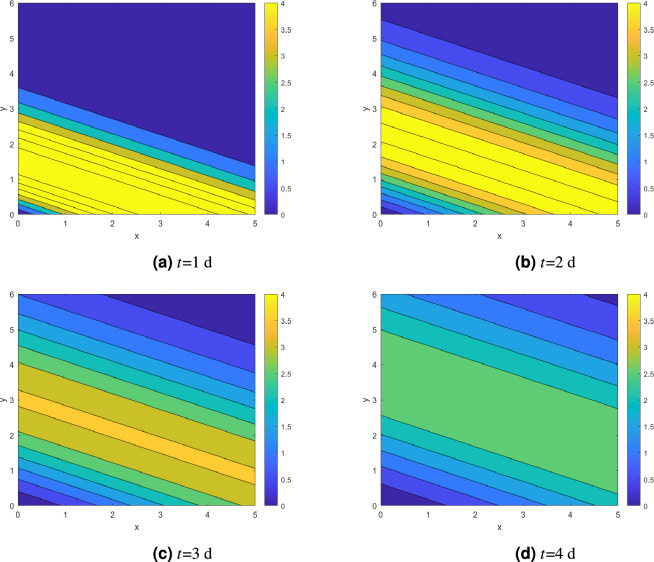
Concentration profiles of heavy metals for *t*=1,2,3,4 d and *R*= 1.5.

Figure 1 is a three-dimensional plot, while Fig. 2 is a two-dimensional contour plot. They respectively depict the distribution of heavy metal concentration at different time points (*t*=1,2,3,4 d). From Fig. 1, it can be observed that the concentration shows an increasing trend until it reaches its peak. After that, the concentration decreases throughout the domain where at earlier time, the decrement is much more faster compared to at later time *t* which is consistent with the observation following Fig. 2. Over time, the concentration in that region gradually spreads toward the surrounding areas. Eventually, it reaches a steady state as the migration process continues.

**Figure 3:**
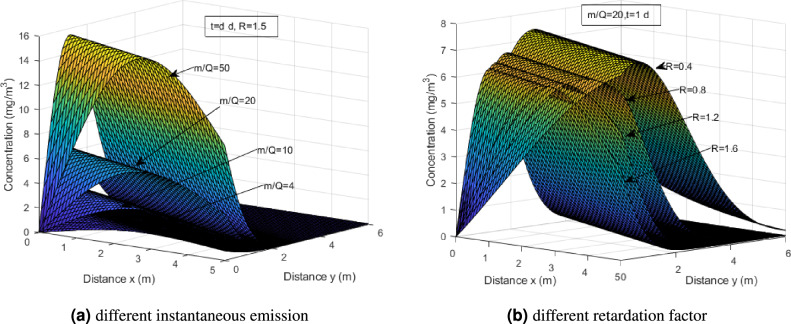
Concentration profiles of heavy metals for different instantaneous emission and retardation factor.

Figure 3a compares the concentration of heavy metal at the same time $t=1$ under different instantaneous emission values. To be specific, *m* is maintained as in Table 1, but the *Q* is evaluated at *Q*=2,5,10,25. The peak values approximately are 15.49, 6.22, 3.13 and 1.27 respectively. From the graph, it can be observed that as the instantaneous emission coefficient increases, the peak concentration of the heavy metal also increases as expected. As the coefficient decreases, the peak concentration of the heavy metal significantly decreases. However, regardless of the coefficient, the concentration eventually reaches an equilibrium state. Figure 3b shows heavy metal concentration for different retardation factor *R*, also at fixed time *t*=1 day. The overall pattern is consistent with our observation following Figs. 1 and 3a, and furthermore, smaller *R* develop a higher concentration at the peak. Based on the information provided by Fig. 3b, it can be inferred that the retardation factor plays a crucial role in the migration and distribution of heavy metal concentration. The retardation factor represents the extent to which the movement of the heavy metal is impeded compared to the flow of the surrounding medium. A smaller retardation factor indicates less hindrance to the movement of the heavy metal, allowing it to migrate more freely and accumulate to higher concentrations at the peak.

### Exponential attenuation of concentration

**Figure 4:**
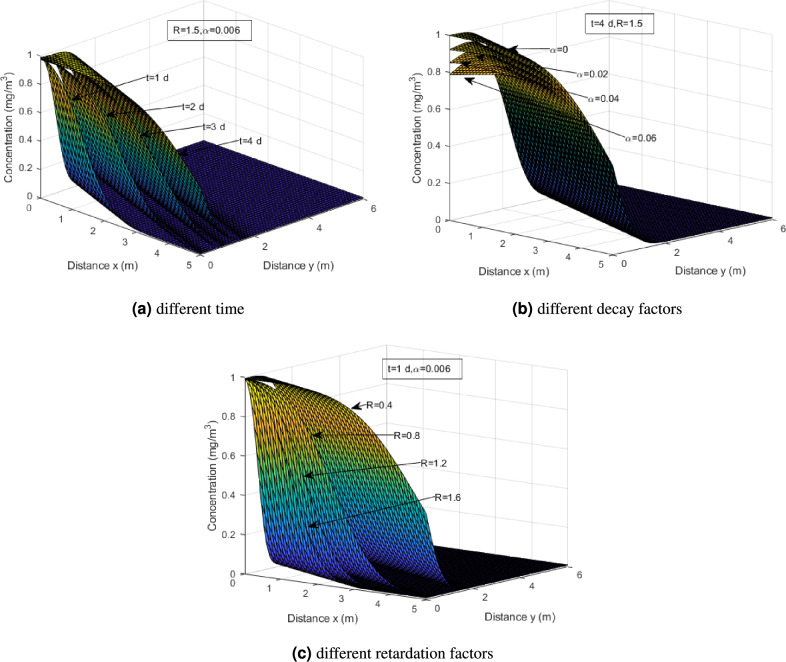
Concentration profiles of heavy metal with exponential decay at different time, decay factors and retardation factors.

The obtained analytical solution of Equation is analysed by plotting the contour plot given in Fig. 4a–c. The parameters are based on previous studies^11^ and^12^ as shown in Table 2.

**Table 2:** Model parameters for exponential attenuation of concentration simulations^11,12^.

$C_0$	$\alpha (s^{-1})$	$k_x (s^{-1})$	$k_y (s^{-1})$	*R*	*a*	*b*	*u*(*cm*/*s*)	*v*(*cm*/*s*)	$C_i (mg/m^3)$
0.01	0.006	0.01	0.02	1.5	0.1	0.2	0.75	0.075	0.05

Figure 4a illustrates the concentration over time in the context of heavy metal transport. It can be seen that the curves demonstrate a gradual decrease in concentration. At $x=0$ and $y=0$, the values of concentration are 0.994, 0.988, 0.983 and 0.979 respectively for *t*=1,2,3 and 4. This is consistent with the boundary condition, such that the attenuation is exponential in nature, indicating a diminishing rate of decrease over time. At earlier times, the concentration reduction is more rapid, resulting in steeper slopes. By examining the relative positions of the curves, one can observe the cumulative effect of exponential attenuation over time. This visual representation provides insights into the temporal dynamics of heavy metal transport and the gradual reduction in concentration due to time and exponential decay processes. In Fig. 4b, it is aimed to investigate the effect of the decay coefficient to the concentration profile by changing the values of $\alpha$, at a specific time $t=4$ days and $R=1.5$. It is observed that the concentration shows a decreasing trend. Also, the plots display 4 curves that start at a high concentration value and gradually decrease towards zero at steady-state concentration. Furthermore, the curve exhibits an exponential decay pattern where the concentration decreases rapidly at first, and then the rate of decrease gradually slows down. By comparing the heavy metal concentrations with different decay coefficients, it can be observed that as the decay coefficient increases, the peak concentration of the particles gradually decreases. This indicates that the decay of the injected concentration has a significant impact on heavy metal migration.

The effects of different retardation factors are studied based on Fig. 4c at specific time $t=1$, when $\alpha$ is 0.006. From Fig. 4c, it showcases that for higher retardation factors, the concentration of heavy metals will exhibit a significant decrease compared to lower retardation factors. This indicates that a greater degree of retardation slows down the transport and reduces the concentration of heavy metals in the soil. Conversely, lower retardation factors result in a less pronounced decrease in concentration. The concentration of heavy metals remains relatively higher as the transport processes are less hindered by retardation effects.

## Conclusion

In this paper, analytical solutions for the two-dimensional migration of heavy metal under instantaneous emission and exponential attenuation of concentration is derived by considering new adsorption term which taking into account the desorption effect given by the rate of change of the concentration. The results are interpreted to present a comprehensive analysis of heavy metal transport in porous media, considering various factors such as time, emission conditions, exponential decay, and retardation effects. The proposed two-dimensional transport model provides valuable insights into the behavior of heavy metal and their concentrations in soil.

The analysis demonstrates that heavy metal concentrations exhibit a significant decrease over time. Moreover, the concentration response is influenced by the instantaneous emission coefficient, with the peak concentration of heavy metals greatly affected by this value. Furthermore, the study reveals the impact of retardation factors on heavy metal concentrations. It is observed that as the retardation factors decrease, there is an increase in the concentration of heavy metals. This finding underscores the significance of accurately characterizing and accounting for retardation processes when predicting and managing heavy metal contamination.

The analysis also examines the exponential attenuation of concentration over time. The concentration profiles depict the gradual reduction in heavy metal concentrations, with steeper slopes initially and slower attenuation rates as time progresses. The curves illustrate the cumulative effect of exponential decay on heavy metal concentrations, offering insights into the temporal dynamics of transport.

In comparison to prior studies, Arora et al.^24^ only discussed how the heavy metal adsorbed and removal which can not provide some necessary information to environment researchers. Aral et al.^8^ have explored instantaneous and exponentially decaying concentration scenarios, but the majority of these investigations have been confined to one-dimensional settings. Other examples^5,25^ only consider the adsorption term and neglect the desorption effect. The findings of this study underscore the role of emission type in determining peak concentrations of heavy metals. When heavy metals are introduced instantaneously, the peak concentration occurs at a distance of approximately 1–3 m from the point of emission on the first day. In contrast, for emissions following an exponential attenuation pattern, the peak concentration happens near the emission point. To sum up, this study provides understanding of heavy metal transport in porous media, considering factors such as time, emission conditions, exponential decay, and retardation effects. The findings contribute to our knowledge of heavy metal behavior and offer valuable insights for environmental remediation strategies. By incorporating these insights into environmental models and management plans, scientists and policymakers can make informed decisions to mitigate heavy metal pollution and safeguard ecosystems and human health.

## Data Availability

All data generated or analyzed during this study are included in this published article.
